# Potentiating humoral and cellular immunity using a novel hybrid polymer-lipid nanoparticle adjuvant for HBsAg-VLP vaccine

**DOI:** 10.1186/s12951-023-02116-6

**Published:** 2023-11-22

**Authors:** Xuhan Liu, Qiuxia Min, Huiping Song, Aochun Yue, Qin Li, Qing Zhou, Wei Han

**Affiliations:** 1 Department of Emergency Medicine, Shenzhen University General Hospital, Shenzhen University Clinical Medical Academy, Shenzhen University, No. 1098 Xueyuan Avenue, Shenzhen, 518000 Guangdong China; 2 Department of Pharmacy, First People’s Hospital of Yunnan Province, Kunming University of Science and Technology, No. 157 Jinbi Road, Kunming, 650034 Yunnan China; 3 First School of Clinical Medicine, Shandong University of Traditional Chinese Medicine, Jinan, China; 4 Centre of Integrated Chinese and Western Medicine, School of Clinical Medicine, Qingdao University, Qingdao, China; 5 The Center for Biomedical Research, Tongji Hospital, Tongji Medical College, Huazhong University of Science and Technology, Wuhan, 430030 China

**Keywords:** Hybrid polymer-lipid nanoparticle, HBV, Vaccine adjuvant, Humoral immunity, Cellular immunity

## Abstract

Aluminium adjuvants are commonly used in vaccines to stimulate the immune system, but they have limited ability to promote cellular immunity which is necessary for clearing viral infections like hepatitis B. Current adjuvants that do promote cellular immunity often have undesired side effects due to the immunostimulants they contain. In this study, a hybrid polymer lipid nanoparticle (HPLNP) was developed as an efficient adjuvant for the hepatitis B surface antigen (HBsAg) virus-like particle (VLP) vaccine to potentiate both humoral and cellular immunity. The HPLNP is composed of FDA approved polyethylene glycol-b-poly (l-lactic acid) (PEG-PLLA) polymer and cationic lipid 1, 2-dioleoyl-3-trimethylammonium-propane (DOTAP), and can be easily prepared by a one-step method. The cationic optimised vaccine formulation HBsAg/HPLNP (w/w = 1/600) can maximise the cell uptake of the antigen due to the electrostatic adsorption between the vaccine nanoparticle and the cell membrane of antigen-presenting cells. The HPLNP prolonged the retention of the antigen at the injection site and enhanced the lymph node drainage of antigen, resulting in a higher concentration of serum anti-HBsAg IgG compared to the HBsAg group or the HBsAg/Al group after the boost immunisation in mice. The HPLNP also promoted a strong Th1-driven immune response, as demonstrated by the significantly improved IgG2a/IgG1 ratio, increased production of IFN-γ, and activation of CD4 + and CD8 + T cells in the spleen and lymph nodes. Importantly, the HPLNP demonstrated no systemic toxicity during immunisation. The advantages of the HPLNP, including good biocompatibility, easy preparation, low cost, and its ability to enhance both humoral and cellular immune responses, suggest its suitability as an efficient adjuvant for protein-based vaccines such as HBsAg-VLP. These findings highlight the promising potential of the HPLNP as an HBV vaccine adjuvant, offering an alternative to aluminium adjuvants currently used in vaccines.

**Supplementary Information:**

The online version contains supplementary material available at 10.1186/s12951-023-02116-6.

## Introduction

While combating the spread of infectious diseases, vaccination stands as a highly effective method for preventing hospitalization and fatality [1, 2]. Despite most marketed vaccines consisting of attenuated or inactivated pathogens, the trend is shifting towards the design of microbial subunit vaccines based on specific antigenic components from the pathogens. As microbial subunit vaccines do not contain live components of the pathogen, there is no risk of disease transmission, making them safer and more stable than vaccines containing whole pathogens [3]. Among subunit vaccines, virus-like particles (VLPs) formed by self-assembled viral proteins are highly desirable due to their exceptional immunogenicity [4, 5]. The Hepatitis B surface antigen (HBsAg) and human papillomavirus (HPV) vaccines are well-known examples of VLP-based vaccines [6–8]. However, VLP-based vaccines face several interrelated pharmacological challenges, including rapid degradation and clearance, low accumulation in secondary lymphoid organs, and inefficient delivery of antigen to the major histocompatibility complex (MHC) class I antigen processing pathway, which is crucial for inducing a CD8 + T cell response [9–11]. As a result, VLP-based antigens alone may not be immunogenic enough, and often require the addition of appropriate adjuvants to enhance the immune response [12].

Adjuvants are immunomodulatory substances added to vaccines for several benefits, such as enhanced immunogenicity, antigen dose sparing, quicker immune response, prolonged duration of prophylaxis, and less booster vaccinations [13, 14]. Adjuvants act as an antigen reservoir and increase the duration of antigen presentation to immune cells. They also stimulate the recruitment and activation of antigen-presenting cells (APCs), which play a critical role in immunostimulation. APCs process vaccine antigens and transport them to draining lymph nodes, where they promote the proliferation and differentiation of T and B cells [15].

To date, most licensed VLP-based vaccines utilize the classic aluminium adjuvants, such as aluminium hydroxide or aluminium phosphate. These adjuvants are highly effective in enhancing antibody responses as they can adsorb antigens through electrostatic interaction, which helps to maintain the physiochemical properties and stability of the vaccine [16]. When injected, antigens that are bound with adjuvants diffuse more slowly from the site of administration, allowing antigen-presenting cells (APCs) to accumulate for better recognition, processing, and presentation of the antigens. Flach et al. [17] demonstrated that aluminium adjuvant could perturb the DC membrane and induce cell membrane lipid reordering without entering the cell, which further lead to antigen uptake and upregulation of CD4 + T cells. In 2019, global immunization coverage for the three-dose Hepatitis B vaccine reached an impressive 80% [18]. These vaccines are available as combination vaccines or single-antigen versions, with the latter commonly administered to high-risk individuals. The usage of aluminium adjuvants is widespread in recombinant Hepatitis B vaccines. In the United States, PreHevbrio, Recombivax HB, Engerix-B, and Heplislav-B are approved for use, with all except Heplislav-B utilizing aluminium adjuvants [19–24]. Similarly, in the United Kingdom, all three licensed Hepatitis B vaccines incorporate aluminium adjuvants [25]. In China, the recombinant Hepatitis B vaccines, including the Recombinant Hepatitis B Vaccine (Saccharomyces Cerevisiae), Recombinant Hepatitis B Vaccine (Hansenula Polymorpha), and Recombinant Hepatitis B Vaccine (CHO Cell), employ hydrated aluminium hydroxide as an adjuvant [26, 27]. However, aluminium adjuvants are usually associated with adverse reactions such as localized inflammation, pain and tenderness at the injection site, and allergic reactions, immunosuppression, and even teratogenic, carcinogenic, and mutagenic risks [28–33]. In some cases, they have also been linked to neurodegeneration, renal dysfunction, and the increased production of eosinophils and immunoglobulin E due to the propensity of Th2 immune bias [34]. Furthermore, substantial amounts of mechanistic studies have demonstrated that the aluminium salt-based adjuvants stimulate the Th2 immune responses, with minimum or no Th1 responses [35]. It is not ideal for viral diseases that require a Th1-mediated immunity and activation of cytotoxic T cells for protection. Th1-type immune responses can be stimulated by using innate immunostimulants such as CpG oligodeoxynucleotides [36], monophosphoryl lipid A [37], and poly I: C [38], however, immunostimulants may produce adverse reactions such as autoimmune diseases while effectively inducing an immune response.

It has been known that Class I MHC (MHC-I) molecules present primarily endogenous antigens, i.e. antigens that are present in the cytosol and are subject to the cytosolic processing mechanisms that comprise the conventional MHC-I processing pathway, which plays important roles in generating CD8 T cell responses [39]. After phagocytic or endocytic uptake, some exogenous antigens can escape the vacuole system and penetrate into the cytosol, accessing the conventional MHC-I antigen processing mechanisms [40]. Thus, it is possible for adjuvants to deliver exogenous antigens to stimulate CD8 T cell responses by mechanisms that may contribute to the substitution of MHC-I processing pathways. The cationic delivery system with “sponge effect” has the potential to allow for the exogenous antigen to escape from the endosomes and enter the cytosol for inducing CD8 + T cell responses via the MHC-I pathway [41].

Over the past few decades, substantial research efforts have been dedicated to the development of alternative vaccine adjuvants. The development and synthesis of adjuvants to regulate the immune system and enhance vaccine efficacy have been widely studied for the improvement of vaccine design and treatment of infectious diseases. The properties of adjuvants depend largely on particle size [42], hydrophobicity [43], administration route [44], antigen release kinetics [45], and surface charge [46]. For example, positive-charged adjuvants like liposomes, cationic polymers, or emulsions interact well with negative-charged cell membranes of antigen-presenting cells (APCs), promoting the intracellular uptake of antigens [47, 48]. However, these adjuvants are often composed of multiple components and complicated to prepare, making overall quality control more complex. For instance, chitosan, a cationic polymer, acts as an adjuvant largely based on its structure, amphiphilicity, and surface charge of self-assembled structures [49]. However, the varying quality of chitosan in different batches due to its complicated synthesis can affect the immunogenicity of vaccines and limit its clinical use [50, 51]. There is a pressing need for the development of adjuvants which have a broad-spectrum of safety, good stability, ease of production and use, and can effectively activate humoral and cellular immune responses with no adverse reactions.

**Fig. 1:**
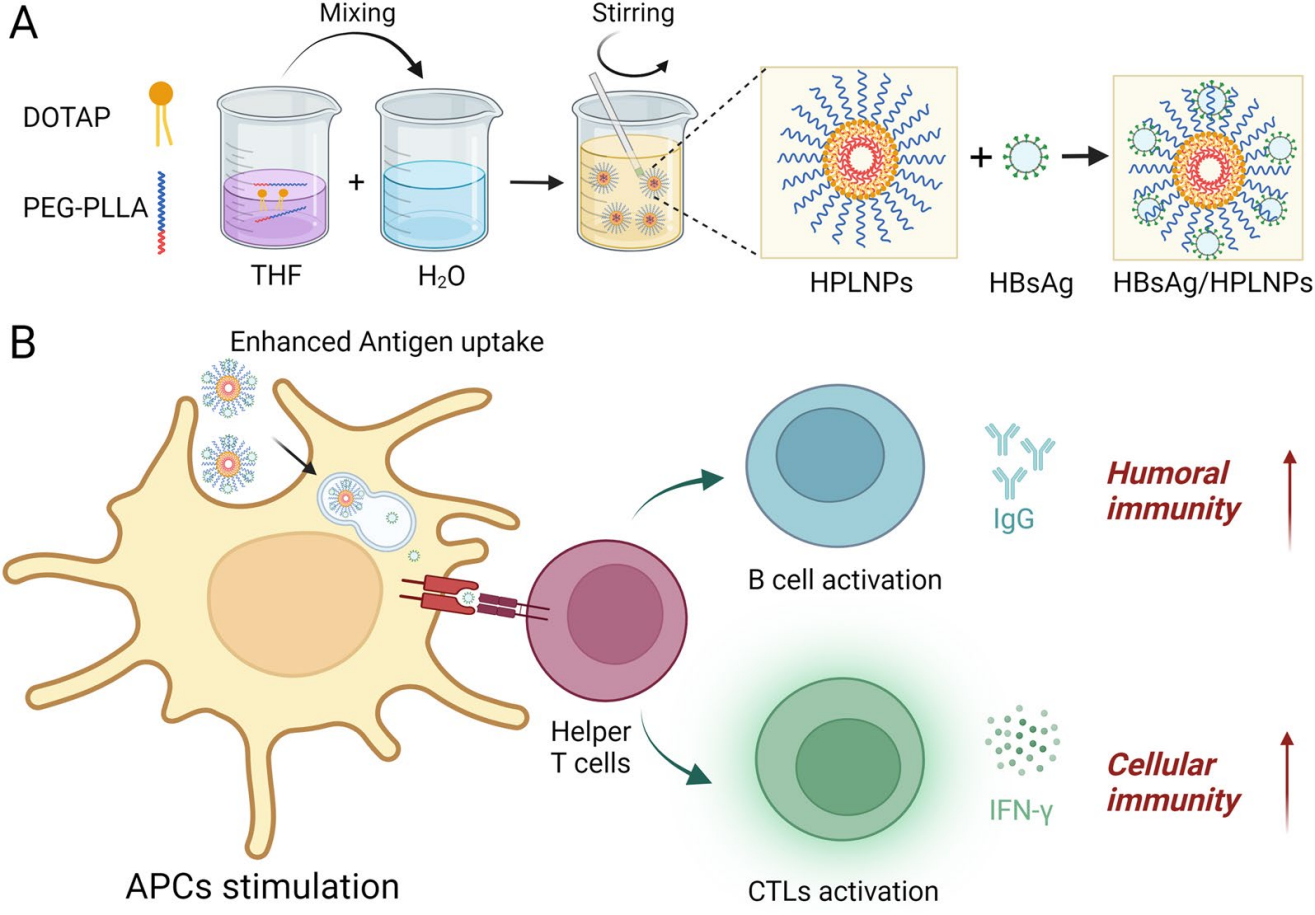
**A** Schematic illustration depicting the preparation and self-assembly structure of HPLNPs, **B** as well as the potential multifaceted immune-stimulatory effects induced by the HBsAg/HPLNP vaccine formulation. Created by BioRender

Based on our previous work, hybrid nanoparticles provide a convenient approach for multiple functionalities and regulating properties [52, 53]. The structures and properties of hybrid nanoparticles can be easily regulated by changing the mixing ratios of different components rather than complicated synthesis. Cationic hybrid nanoparticles may be an effective vaccine adjuvant to potentiate the immune response, as they can delivery antigens and enhance the cellular uptake of antigens by APCs through electrostatic absorption. In light of the challenges posed by current vaccine adjuvants, we aim to develop a cationic hybrid polymer lipid nanoparticle (HPLNP) as an efficient vaccine adjuvant with multifaceted immune responses (Fig. 1B). The HPLNP is composed of two FDA-approved materials: polyethylene glycol-b- poly (L-lactic acid) (PEG-PLLA) polymer and cationic lipid 1,2-dioleoyl-3-trimethylammonium-propane (DOTAP). As shown in Fig. 1A, the HPLNP can be prepared by a simple one-step method based on several minutes of mixing, stirring and organic solvent evaporation. The physicochemical properties of HPLNPs can be preciously controlled by regulating the mixing ratios of polymer and lipid. The size distribution, PDI, and zeta potential of HPLNPs were analysed through dynamic light scattering, and the morphology was confirmed through transmission electron microscopy (TEM). The HBsAg-VLP was used as a model antigen and mixed with the HPLNP to create vaccine formulations. Flow cytometry was applied to optimise the vaccine formulation through analysing the intracellular uptake of antigen by APCs. Finally, the in vivo immunisation of the optimised vaccine formulation was carried out. The antigen depot effect and the lymph node drainage of the optimised vaccine formulation were studied by the small animal in vivo imaging. The humoral responses of different formulations were compared by analysing the serum anti-HBsAg IgG concentration at specific time points. The IgG2a/IgG1 ratio was calculated to estimate the Th1/Th2 immune response trend. The secretion of cytokines in serum was evaluated by Elisa kit. Meanwhile, the activation of B cells and T cells in the spleen or lymph nodes was tested by flow cytometry. For biosafety assessment, the immunohistochemical staining of different organs and intramuscular injection sites post prime immunisation were finally analysed and the mice body weights were monitored during the immunisation.

## Materials and methods

### Materials

mPEG_5k_-PLLA_10k_ was bought from Tansh Tec Co., LTD (Guangzhou, China), tetrahydrofuran (THF), 4% (w/v) paraformaldehyde solution, fluorescein isothiocyanate (FITC), 4’,6-diamidino-2-phenylindole (DAPI), Triton X-100 were purchased from Sigma-Aldrich. Cy5.5-labeled BSA was purchased from Shanghai Yuanye Biotechnology Co. LTD. 1, 2-dioleoyl-3-trimethylammonium-propane (chloride salt) DOTAP was bought from Avanti Polar Lipids. CCK8 assay was obtained from Abcam (Cambridge, UK) Trypsin–EDTA (0.25%, w/v), fetal bovine serum (FBS) and penicillin–streptomycin (100×) were bought from Gibco (CA, USA). Phosphate buffered saline (PBS), RPMI-1640 medium and high glucose Dulbecco's modified eagle medium (DMEM) were obtained from Hyclone lab (UT, USA). Fixation/Permeabilization solution and Perm/Wash buffer were purchased from Nuowei Biotechnology Co. LTD (Beijing, China). Sulfo-Cyanine5.5 (Cy5.5) fluorescently labelled antibody protein/kit and fluorescein isothiocyanate (FITC) fluorescently labelled antibody protein/kit were purchased from Abcam (Cambridge, UK). Antibodies of PerCP/Cy5.5-CD3, ER780-CD8a were obtained from Elabscience Biotechnology Co. LTD. Antibodies of Ms CD4 FITC GK1.5, Ms CD8a PerCP-Cy5.5 (53–6.7), Ms CD19 APC-Cy7 1D3, Ms CD69 APC H1.2F3 and Ms CD178 PE MFL3 were brought from Becton, Dickinson and Company (New Jersey, USA). Mouse ELISA Kits of Anti-HBsAg IgG, Anti-HBsAg IgG1 and Anti-HBsAg IgG2a were obtained from Shanghai Runyu Biotechnology Co. LTD (Shanghai, China). Mouse ELISA Kits of IL-4, IL-6, IFN-γ and Gzms-B were purchased from Beyotime Biotechnology Co. LTD (Shanghai, China). RAW264.7 cells were obtained from the ATCC (American Type Culture Collection, Wesel, Germany). Sheep blood was purchased at TCS Biosciences (Buckinghamshire, UK). HBsAg-VLP antigen was both kindly gifted by AIM Honesty Biopharmaceutical Co., Ltd (AIM, Dalian, China). Al adjuvant was obtained from Miragen (Delaware, USA). FITC anti-mouse MHC II (I-A/I-E) antibody was purchased from SALMART (Shenzhen, China). MACS^®^ BSA Stock Solution was purchased from Miltenyi Biotec Technology & Trading (Shanghai) Co., Ltd. The mouse spleen lymphocyte separator kit was obtained from Solarbio (Beijing, China).

### Preparation and characterisation of HPLNP and HBsAg/HPLNP formulations

The HPLHNs were prepared by a simple one-step method presented in Fig. 1A [54]. Typically, 650 µg PEG_5k_-PLLA_10k_ and 250 µg DOTAP were co-dissolved in 0.5 mL THF, followed by mixing with 1 mL of water. The mixture was kept stirring in a ventilation cabinet and the HPLNPs were obtained until THF was evaporated. The size distribution and zeta potentials of HPLNPs were tested by dynamic light scattering (DLS). TEM was used to observe the morphology of HPLNPs and HBsAg/HPLNP (w/w = 1/600) formulation. Various HBsAg/HPLNP formulations were prepared by mixing the HBsAg and HPLNPs with different mass ratios directly and being incubated for 30 min, and their size distribution and zeta potentials were also evaluated by DLS. A UV–visible spectrophotometer was employed to examine the UV absorption spectra of the HBsAg, HPLNP, and HBsAg/HPLNP samples. The concentration of HBsAg was 0.05 mg ml^−1^, the concentration of HPLNP was 0.27 mg ml^−1^, and in the HBsAg/HPLNP sample, the concentrations of HBsAg and HPLNP matched those in their respective individual samples. The scanning spectra ranged from 190 to 300 nm. Additionally, UV absorption spectra were re-measured for the HBsAg/HPLNP sample after a 7-day storage period at 4 °C. Besides, the size distribution and zeta potential of the HBsAg/HPLNP (w/w = 1/600) formulation were also tested by DLS after storage in PBS at 4 °C for 7 days.

### In vitro antigen release

The Cy5.5-labeled Bovine Serum Albumin (Cy5.5-BSA) was utilized as the model antigen to study the in vitro antigen release profile. The BSA/HPLNP formulation with a weight ratio of 1/600 was prepared using the previous method described for the HBsAg/HPLNP (w/w = 1/600) formulation in "Preparation and characterisation of HPLNP and HBsAg/HPLNP formulations" section. Then the BSA/HPLNP (w/w = 1/600) formulation, containing 40 µg of BSA, was incubated in a 100 kDa molecular weight cut off dialysis bag (Yuanye Biotechnology Co., Ltd, Shanghai, China) in PBS (pH 7.4) at 37 °C for 72 h. The accumulated release of BSA from the BSA/HPLNP formulation was analysed at predetermined time intervals by measuring the fluorescence intensity of the release media using a fluorescence spectrophotometer (Yidian 970CRT, Shanghai, China), resulting in the generation of the corresponding release curve. The BSA/Al group, which was prepared by mixing BSA and aluminium adjuvant at a mass ratio of 1/25 as per the manufacturer’s instructions, was used as a control, along with the BSA group.

### Cell culture

RAW264.7 cells were cultured in high glucose DMEM medium supplemented with 10% (v/v) FBS and 1% (v/v) penicillin–streptomycin (100×) in a humidified atmosphere with 5% CO_2_ at 37 °C.

### Cytotoxicity and haemolysis of HPLNPs

The cytotoxicity of HPLNPs with different concentrations on RAW264.7 cells or spleen lymphocytes was analysed using a CCK8 assay. To extract the spleen lymphocytes, mice were euthanized, and the spleen was aseptically excised and transferred to a petri dish with the chilled MACS^®^ BSA stock solution consisting of PBS and 10% (v/v) BSA. The spleen tissue was pressed through a strainer using a syringe barrel to obtain a single-cell suspension. After centrifugation with the mouse spleen lymphocyte separator kit according to the manufacturer’s protocol, the spleen lymphocytes were obtained. RAW264.7 cells or the spleen lymphocytes were seeded in a 96-well plate at a density of 5 × 10^4^ cells/well. After incubation for 24 h, cells were treated with various concentrations of HBsAg, HBsAg/Al or HBsAg/HPLNP (w/w = 1/600) formulations in a completed DMEM medium for 24 h. Then, 10 μL of CCK8 solution was added to each well. After further incubation for 4 h, the absorbance of each well at 450 nm was measured using a Spectrofluorometer (GloMax^®^ Discover Microplate Reader, Promega, USA). The cytotoxic effect was determined from the absorbance readings.

The haemolysis of HPLNPs was also investigated. Briefly, defibrinated sheep erythrocytes (RBCs) were centrifuged at 1500*g* for 10 min at 4 °C and washed with PBS for three times. The cell pellets were re-suspended into a 5% (v/v) erythrocyte suspension with PBS. The HPLNPs was added into the RBC suspension with a final concentration at 0.5 mg mL^−1^. Al adjuvant was separately added into the RBC suspension at the same concentration for comparison. After incubation at 37 °C for 1 h, the RBC suspension was centrifuged, and 100 μL of the supernatant was transferred to a 96-well plate. The RBC suspension treated with deionized water or PBS was used as the positive or negative control, respectively. The absorbance (A) was measured at 540 nm using a spectrofluorometer (GloMax^®^ Discover Microplate Reader, Promega, USA). The relative haemolysis was calculated according to the following equation:

$$\mathrm{Relative haemolysis\,}\left(\mathrm{\%}\right)= \frac{{\mathrm{A}}_{\mathrm{S}}-{\mathrm{A}}_{\mathrm{NC}}}{{\mathrm{A}}_{\mathrm{PC}}-{\mathrm{A}}_{\mathrm{NC}}}$$

where the A_S_, A_NC_ and A_PC_ represent the absorbance of the sample, the negative control and the positive control respectively.

### Cellular uptake of HBsAg/HPLNP formulation by macrophages

FITC-labelled HBsAg was obtained according to the protocol of fluorescein isothiocyanate (FITC) fluorescently labelled antibody protein/kit. Flow cytometry was utilized to examine the cellular uptake of the HBsAg/HPLNP formulations. The HBsAg/HPLNP formulations were prepared by mixing the FITC-conjugated HBsAg and HPLNP in varying HBsAg/HPLNP mass ratios, ranging from 1/20 to 1/1600. The size distribution and zeta potential of these formulations were analysed using DLS.

RAW264.7 cells were seeded in the 6-well plate at the density of 4 × 10^5^ per well for 24 h. After replacing the culture medium into fresh serum-free medium, the HBsAg or various HBsAg/HPLNP formulations with different mass ratios were added in at a concentration of 1 µg mL^−1^ of HBsAg. Following a 4-h incubation period, the cells were washed with PBS for three times and collected for analysing by flow cytometry. The percentage of FITC-positive cells and the mean fluorescence intensity (MFI) were both evaluated. Meanwhile, 2 × 10^5^ of RAW264.7 cells were also seeded in the confocal dish for 24 h. The HBsAg or HBsAg/HPLNP (w/w = 1/600) formulation was added at the concentration of 1 µg mL^−1^ of HBsAg and incubated for another 4 h. Then the cells were washed with PBS for three times and incubated with 5 µg mL^−1^ of DAPI for 10 min. After washing with PBS and fixed with 4% paraformaldehyde, the cells were observed under a fluorescence confocal microscope (Leica SP8 Inverted) to investigate the intracellular uptake of HBsAg.

RAW264.7 cells were cultured in 12-well plates at a density of 4 × 10^5^ cells per well for 24 h. Subsequently, HBsAg, HBsAg/Al or HBsAg/HPLNP were introduced at a concentration of 0.5 μg HBsAg per well, followed by a 24-h incubation period. Following this, cells were washed with PBS, collected, and subjected to staining with FITC anti-mouse MHC II (I-A/I-E) antibody for 15 min at room temperature. After PBS washing, flow cytometry analysis (Sparrow, China) was employed to assess the percentage of MHC II positive cells.

### Animals

6–8 weeks-old female C57BL/6 mice used for animal experiments were purchased from SPF (Beijing) Biotechnology Co., Ltd. The mice were maintained under specific pathogen-free condition at the animal facility in the Key Lab, Shen Zhen University General Hospital.

### Injection site antigen depot and lymph nodes drainage

The Cy 5.5-labelled HBsAg was prepared according to the protocol of Cy5.5 fluorescently labelled antibody protein/kit for analysing the antigen depot at the injection site. 6–8 weeks-old female C57BL/6 mice were grouped (n = 4) and intramuscularly injected at the right back leg with HBsAg, HBsAg/Al or HBsAg/HPLNP (w/w = 1/600) formulations at the dose of 1 µg HBsAg per mouse. After intramuscular administration, the fluorescence graphs of the injection site were collected through in vivo imaging system FX Pro (Kodak), at 12, 24, 48 and 72 h. The mean fluorescence intensity graph of mice at the injection site in each group was obtained at different time points. After intramuscular administration, the fluorescence signals of mesenteric lymph nodes were also collected at 12, 24, 48 and 72 h. The mean fluorescence intensity at the injection sites and mesenteric lymph nodes was calculated for different groups to compare the efficacy of various vaccine formulations in terms of antigen depot effect and lymph nodes drainage.

### Activation of lymphocytes in lymph nodes

The mice were divided into HBsAg, HBsAg/Al and HBsAg/HPLNP (w/w = 1/600) groups (n = 5) and intramuscularly injected with various formulations at the dose of 1 µg HBsAg per leg. After 18 h, the mice were sacrificed and the mesenteric lymph nodes were collected. The lymphoid tissue suspension can be prepared by initially cleaning the tissue with PBS and subsequently cutting it into small pieces (1–2 mm^3^), followed by using a tissue grinder equipped with a sandpaper mesh to grind the lymph nodes until complete disintegration. This process is repeated until all the lymph nodes are uniformly homogenised. The resulting homogenate is then transferred into a centrifuge tube, and the serum-free RPMI-1640 medium is added to prepare the lymphoid tissue suspension. To obtain a single-cell suspension, the lymphoid tissue suspension is aspirated using a 5 mL syringe with a 21G needle, filtered through a 40 μm-cell strainer, and collected into a 15 mL centrifuge tube. The desired single-cell suspension was applied for further downstream applications. To prepare the cells for intracellular staining, the cell pellet is resuspended in 250 µL of fixation/permeabilization solution per tube and incubated at 4 °C for 30 min in the dark. After fixation and permeabilization, the cells are centrifuged at 450 g for 5 min, and 1 mL of 1 × Perm/Wash™ buffer is added, followed by incubation for 15 min. Next, the cells are centrifuged at 450 g for 5 min at 4 °C, and the fixed and permeabilized cells are resuspended in 100 µL of 1 × Perm/Wash™ buffer. Subsequently, the desired fluorescently conjugated antibody including PerCP/Cy5.5-CD3, ER780-CD8a, Ms CD4 FITC GK1.5, Ms CD19 APC-Cy7 1D3 and Ms CD69 APC H1.2F3 were added at a predetermined optimal concentration, and the mixture is vortexed and incubated for 40–45 min at 4 °C. The cells were analysed by flow cytometry for detecting of activation of B cells and T cells in lymph nodes.

#### In vivo immunisation

The immunisation schedule was shown in Fig. 6A. The study involved testing various vaccine formulations using groups of six mice each. The HBsAg/Al formulation was prepared by mixing with HBsAg and Al adjuvant at a mass ratio of 1/25, following the manufacturer’s guidelines. The optimal mass ratio for the HBsAg/HPLNP formulation was determined to be 1/600. Mice were randomly grouped into four groups (PBS, HBsAg, HBsAg/Al, HBsAg/HPLNP). Each mouse was administered a 50 μL injection of the formulation, at a dose of 1 µg of HBsAg per leg. Boost injections were carried out on Day 14 and Day 28, respectively. The blood was collected at predetermined time points from the orbit venous plexus and was stationarily placed at room temperature for 4 h, before being centrifuged at 10,000*g* for 10 min to obtain the serum. The serum anti-HBsAg IgG concentration was tested on Day 21, Day 35 and Day 42 by ELISA Kits according to the manufacturer’s protocol. The concentrations of serum anti-HBsAg IgG1 and anti-HBsAg IgG2a were also studied on Day 42. Cytokines including IL-4, IL-6, IFN-γ and Gzms-B in serum on Day 42 were also measured by ELISA Kits. Meanwhile the body weights of the mice were monitored throughout the immunisation period.

#### Evaluation of intracellular IFN-γ secretion by splenocytes

After immunisation, the mice were sacrificed on Day 42, and the spleens were extracted and homogenized using a 40 μm-cell strainer. The splenocytes were then separated from the erythrocytes and washed with RPMI 1640 before being resuspended in a complete medium for further experiments. Antibody was coated onto pre-wet PVDF plates under sterile conditions and incubated overnight at 4 °C. The plates were washed for five times, and 200 μL of complete medium was added and incubated for 30 min. Afterwards, 5 × 10^5^ splenocytes were added to each well, and HBsAg (5 mg mL^−1^) was added to re-stimulate the splenocytes for 24 h at 37 °C. The process involved washing the plates and then incubating them with the detection antibody for a period of 2 h. Following this, the plates were washed again and incubated with streptavidin-ALP for 1 h. Subsequently, substrate (BCIP/NBT) was added to initiate spot development in the dark. After 10 min of development, tap water was added to halt colour development, and the plates were left to dry. Finally, the spots were analysed using the ChampSpot Elispot II system.

#### Splenocyte proliferation in vitro

The splenocyte proliferation in vitro was assessed using the CCK8 assay, following the manufacturer’s instructions. In brief, 5 × 10^5^ splenocytes were seeded into a 96-well plate with or without stimulation of 5 μg mL^−1^ HBsAg for 24 or 48 h. After incubation, 10 µL of CCK8 solution in fresh culture medium was added and incubated at 37 °C for another 4 h. The absorbance was measured at 450 nm. The splenocyte proliferation was determined by dividing the absorbance of stimulated cultures by that of non-stimulated cultures, while the absorbance of the medium was used as the background.

#### Detection of the splenocyte activation

The splenocytes were collected as previously described. Following restimulation with 5 μg mL^−1^ HBsAg in vitro for 48 h, the splenocytes were washed and labelled with Ms CD4 FITC GK1.5, Ms CD8a PerCP-Cy5.5 (53–6.7) and Ms CD19 APC-Cy7 1D3 to distinguish T, B cells, respectively. Additionally, Ms CD69 APC H1.2F3 and Ms CD178 PE MFL3 were used to detect the activation of B and T cells, respectively. After a 30 min incubation at 4 °C, the cells were washed and suspended in 500 μL of PBS buffer before being analysed by flow cytometry.

#### Histologic analysis

Mice were euthanized 24 h after the first intramuscular injection. Samples of the injection site muscle and key organs were collected and processed. They were fixed in 4% paraformaldehyde for 24 h, embedded in paraffin wax, and sliced into 8-mm sections for Haematoxylin and Eosin (H&E) staining. The sections were then examined using a Leica DM3000 optical microscope and photographed.

## Results and discussion

### Preparation and characterisation of HPLNPs

**Fig. 2:**
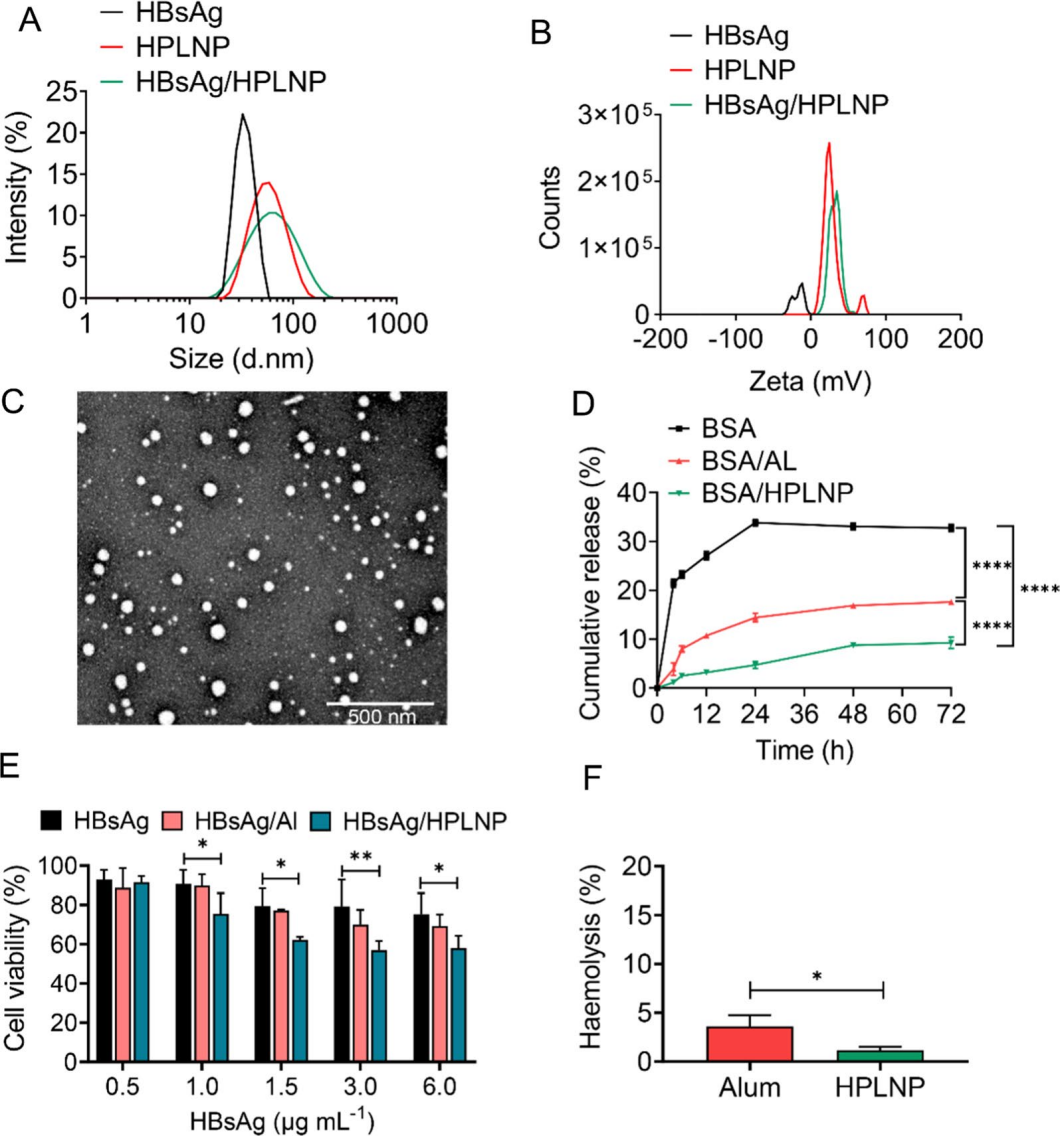
**A** The average size distribution and (**B**) zeta potential of HBsAg, HPLNP and HBsAg/HPLNP (w/w = 1/600) formulation detected by DLS. **C** The TEM graph of the HPLNP. **D** Cumulative release of BSA from different formulations including BSA, BSA/Al and BSA/HPLNP (w/w = 1/600) in PBS at pH 7.4 for 72 h. **E** The cell viability of RAW264.7 cells after being treated with HBsAg, HBsAg/Al or HBsAg/HPLNP (w/w = 1/600) formulation at different concentrations of HBsAg for 24 h. **F** The haemolysis of alum adjuvant and HPLNPs at the same concentration of 0.5 mg mL^−1^. *p < 0.05, **p < 0.01, ****p < 0.0001. Student’s t-test, Two-way ANOVA test or Tukey's multiple comparisons test was used for statistical analyses

**Fig. 3:**
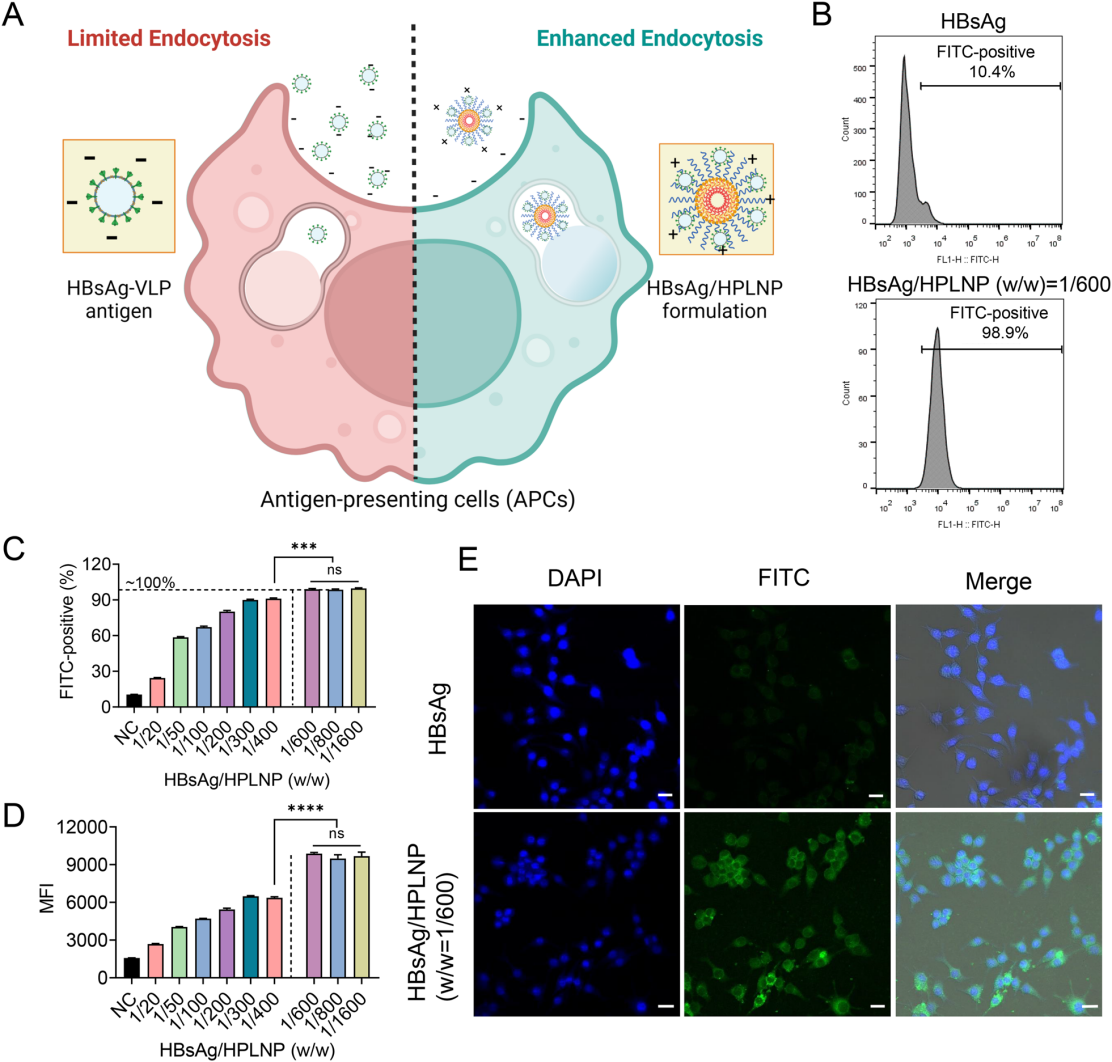
**A** Schematic graph of the cellular uptake of the HBsAg antigen or the cationic HBsAg/HPLNP formulation by APCs. (Created with BioRender.com) **B** The percentage of FITC-positive cells after incubation with HBsAg or HBsAg/HPLNP (w/w = 1/600) formulation for 4 h by flow cytometry. **C** The percentage of cells positive for FITC and (**D**) the mean fluorescence intensity (MFI) in cells were measured by flow cytometry after incubation with HBsAg/HPLNP formulations with various HPLNP/HBsAg weight ratios ranging from 0 to 1600 at a HBsAg concentration of 1 µg mL^−1^ for 4 h. **E** Intracellular fluorescent images were obtained by confocal microscopy after incubation with HBsAg or HBsAg/HPLNP (w/w = 1/600) formulation at a HBsAg concentration of 1 µg mL^−1^ for 4 h (Bar = 20 μm). One-way ANOVA was used for statistical analyse

The HPLNPs were prepared by a simple one-step method based on several minutes of mixing, stirring and organic solvent evaporation as shown in Fig. 1A. After combining HBsAg and HPLNP at different mass ratios, several HBsAg/HPLNP formulations were obtained. Among them, the HBsAg/HPLNP (w/w = 1/600) demonstrated the highest antigen uptake without an excess of HPLNP, as shown in Fig. 3C of "Cellular uptake of vaccine formulations" section. Therefore, the HBsAg/HPLNP (w/w = 1/600) formulation was selected as the representative one for characterisation here. The size distribution and zeta potential of HBsAg, HPLNP and HBsAg/HPLNP (w/w = 1/600) have been shown in Fig. 2A, B. The average size of the HPLNPs was 53.3 ± 0.2 nm in diameter, with a low polydispersity index (PDI) of 0.32 ± 0.01 in Additional file 1: Table S1. The HPLNPs had a positive surface charge, with an average zeta potential of 27.9 ± 1.7 mV. The morphology of the HPLNPs was observed through transmission electron microscopy (TEM) in Fig. 2C, which revealed a uniform size distribution around 50–60 nm, consistent with the DLS data. After mixing with HBsAg antigen (35.9 ± 0.8 nm, − 17.7 ± 1.6 mV), HBsAg/HPLNP formulations with different HBsAg to HPLNP mass ratios showed increased size distribution (around 57–75 nm) and positive surface charge (around 17–37 mV) in Additional file 1: Table S2. The HBsAg/HPLNP (w/w = 1/600) formulation exhibited an average size distribution around 56.7 ± 0.3 nm, which was slightly larger than that of the HPLNPs. The zeta potential of the HBsAg/HPLNP (w/w = 1/600) formulation was measured to be 33.6 ± 0.7 mV as indicated in Additional file 1: Table S1. This value was lower than the zeta potential of the HPLNPs alone, but still positively charged, which is beneficial for the uptake of the formulation by APCs. Additional file 1: Fig. S1 presented the UV absorption characteristics of HBsAg and HPLNP. HBsAg exhibited an absorption peak at 193 nm with an absorbance of 1.96, while HPLNP displayed a distinctive peak at 196 nm with an absorbance of 2.67. When HBsAg and HPLNP were combined in the HBsAg/HPLNP sample, a significant alteration in the UV absorption curve emerged, notably evident by the peak shift to 199–3 nm rightward compared to HPLNP’s peak. The absorbance at 199 nm was 2.93, notably different from the sum of their individual absorbance values (3.59). These outcomes strongly support the successful adsorption of HBsAg onto HPLNP’s surface, as indicated by the distinctiveness of the new curve compared to the additive curve (dashed curve). Notably, the stability of the HBsAg/HPLNP formulation was maintained after storage at 4 °C for 7 days, as evidenced by the minimal deviation from the freshly prepared sample. In Additional file 1: Fig. S2, the size distribution and zeta potential of the HBsAg/HPLNP formulation remained consistent at approximately 57.1 ± 0.2 nm and 33.5 ± 1.3 mV, respectively, after the 7-day storage period at 4 °C. This negligible alteration in comparison to the freshly prepared sample, with dimensions of approximately 56.7 ± 0.3 nm and a zeta potential of 33.6 ± 0.7 mV, further validated the robust stability of the HBsAg/HPLNP formulation. Additional file 1: Fig. S3 showed the TEM graph of the HBsAg/HPLNP (w/w = 1/600) formulation. It revealed the presence of several prominent small round protrusions on the surface of the larger nanoparticles in the centre, indicating the successful surface adsorption of several smaller particles with a diameter of approximately 30 nm (HBsAg-VLP antigens). This confirmed the effective attachment of HBsAg to the surface of HPLNP.

### In vitro antigen release

One of the mechanisms by which adjuvants improve immune response is by adsorbing the antigen and creating a depot effect at the injection site. The in vitro antigen release result, which used a dialysis bag to simulate antigen release at the injection site, have been shown in Fig. 2D. Here, we used BSA as a model protein-based drug, as it is a commonly used negatively charged protein for release studies [55].The cumulative release of BSA in the BSA group without adjuvants increased over time and reached equilibrium at 33.8% after 24 h. In contrast, the groups with Al or HPLNP adjuvants exhibited much slower antigen release rates than the BSA group, and equilibrium was not reached until 48 h. Furthermore, the maximum cumulative release of BSA at 72 h in the BSA/Al group and the BSA/HPLNP group was about 17.4% and 8.6%, respectively, which was significantly lower than that in the BSA group (****p < 0.0001). Meanwhile, the release of BSA in the BSA/HPLNP group was significantly lower than that in the BSA/Al group (****p < 0.0001). These results demonstrated the effective antigen absorption and depot effect of the HPLNPs. Consequently, the HPLNP showed a slower antigen release rate, increasing the likelihood of antigen presentation and subsequent cellular uptake by APCs. These findings suggest that the HPLNP may offer advantages over the Al adjuvant in terms of its ability to facilitate antigen presentation and cellular uptake.

### Cytotoxicity and haemolysis of HPLNPs

To evaluate the biocompatibility of HPLNPs, cell cytotoxicity and haemolysis, which are typical issues associated with cationic carriers due to their strong interaction with negatively charged cell membranes, were tested. The results indicated that the cell viability of RAW264.7 cells decreased as the concentration of HBsAg/HPLNP formulation increase. While an evident decrease in cell viability was noted in the HBsAg/HPLNP group with increasing HBsAg concentration, no statistically significant disparity was observed between the HBsAg/HPLNP and HBsAg/Al groups in Fig. 2E. Importantly, the cytotoxicity associated with HPLNP remained within acceptable limits and over 50% of cells exhibited sustained viability even at elevated concentrations. Additional file 1: Fig. S5 demonstrated the cell viability of mouse spleen lymphocyte after treatment with different formulations. A similar trend was observed in comparison with the cell viability results obtained with RAW264.7 cells. The trend showed an increase in toxicity of HBsAg/HPLNP on splenocytes with higher concentrations. However, no significant difference was noted when compared to the Al adjuvant. The increased cytotoxicity was due to the positive charge accompanied with the HPLNPs. It is encouraging that HPLNPs had a negligible haemolysis rate of less than 5%, which was significantly lower than that of Al adjuvant (*p < 0.05). These results demonstrated that the HPLNP had low cytotoxicity and favourable biocompatibility, making it possible for further in vivo studies.

### Cellular uptake of vaccine formulations

Antigen cellular uptake by APCs is the basis of the protective immune response mechanism in vaccination. Furthermore, cytoplasmic delivery of antigens is crucial for the induction of antigen-specific immune responses. It is well known that soluble antigens alone are poorly effective in protective immunity, mainly because of quick degradation and insufficient cellular uptake by APCs. Thus, adjuvants can make the vaccine more immunogenic by protecting antigen and promoting its cell uptake by APCs (Fig. 3A). Herein, we formulated vaccine formulations using FITC-HBsAg adsorbed to HPLNPs and investigated the cellular uptake of formulations with different components in RAW264.7 cells using flow cytometry. Due to the positive charge of HPLNPs, HBsAg/HPLNPs formulations had a stronger electrostatic attraction to the negatively charged cell membrane, leading to a significantly higher cellular uptake compared to HBsAg alone. In Fig. 3B, only ~ 10.4% of cells in the HBsAg group were FITC-positive, while nearly all of the cells (~ 98.9%) in the HBsAg/HPLNP (w/w) = 1/600 group were FITC-positive. When the mass ratio of HPLNP to HBsAg reached 600, a significantly higher percentage of FITC-positive cells (***p < 0.001) and intracellular MFI (****p < 0.0001) were observed compared with the HBsAg/HPLNP (w/w = 1/400) group. Meanwhile, the percentage of FITC-positive cells and the intracellular MFI were kept stationary when the mass ratio of HPLNP to HBsAg exceeded 600, indicating the saturation of the adjuvant effect of HPLNP. It convincingly demonstrated that the cellular uptake of HBsAg increases with an increase in the amount of HPLNPs in Fig. 3C, D. The fluorescence confocal microscope images also confirmed that the HPLNP significantly enhanced the cellular uptake of HBsAg when the mass ratio of HPLNP to HBsAg reached 600. This was evident from the higher intracellular fluorescence intensity observed in the HBsAg/HPLNP group as compared to the HBsAg group in Fig. 3E. We postulated that the maximum cellular uptake of the antigen could be achieved using the HPLNP formulation with an optimised adjuvant/antigen mass ratio of around 600. Henceforth, the terms “HBsAg/HPLNP” formulations mentioned hereafter will pertain to the adjuvant/antigen mass ratio of 600, unless explicitly stated otherwise.

Typically, ingested antigens undergo degradation into short peptide fragments within the acidic intracellular environment due to enzymatic activity. This process leads to the formation of MHC II-antigen complexes on APCs, subsequently presented to CD4 + T cells, thus initiating humoral immune responses [56]. After incubation with HBsAg, HBsAg/Al or HBsAg/HPLNP formulation for 24 h, flow cytometry was employed to assess the cell surface expression of MHC II molecules. Additional file 1: Fig. S4 revealed a conspicuously elevated proportion of MHC II positive cells within the HBsAg/HPLNP group, in comparison to the HBsAg (****p < 0.0001) and HBsAg/Al (****p < 0.0001) groups. This disparity exhibited statistical significance, underscoring the capability of the HPLNP adjuvant to significantly augment the antigen presentation process.

### Injection site antigen depot and lymph node drainage

**Fig. 4:**
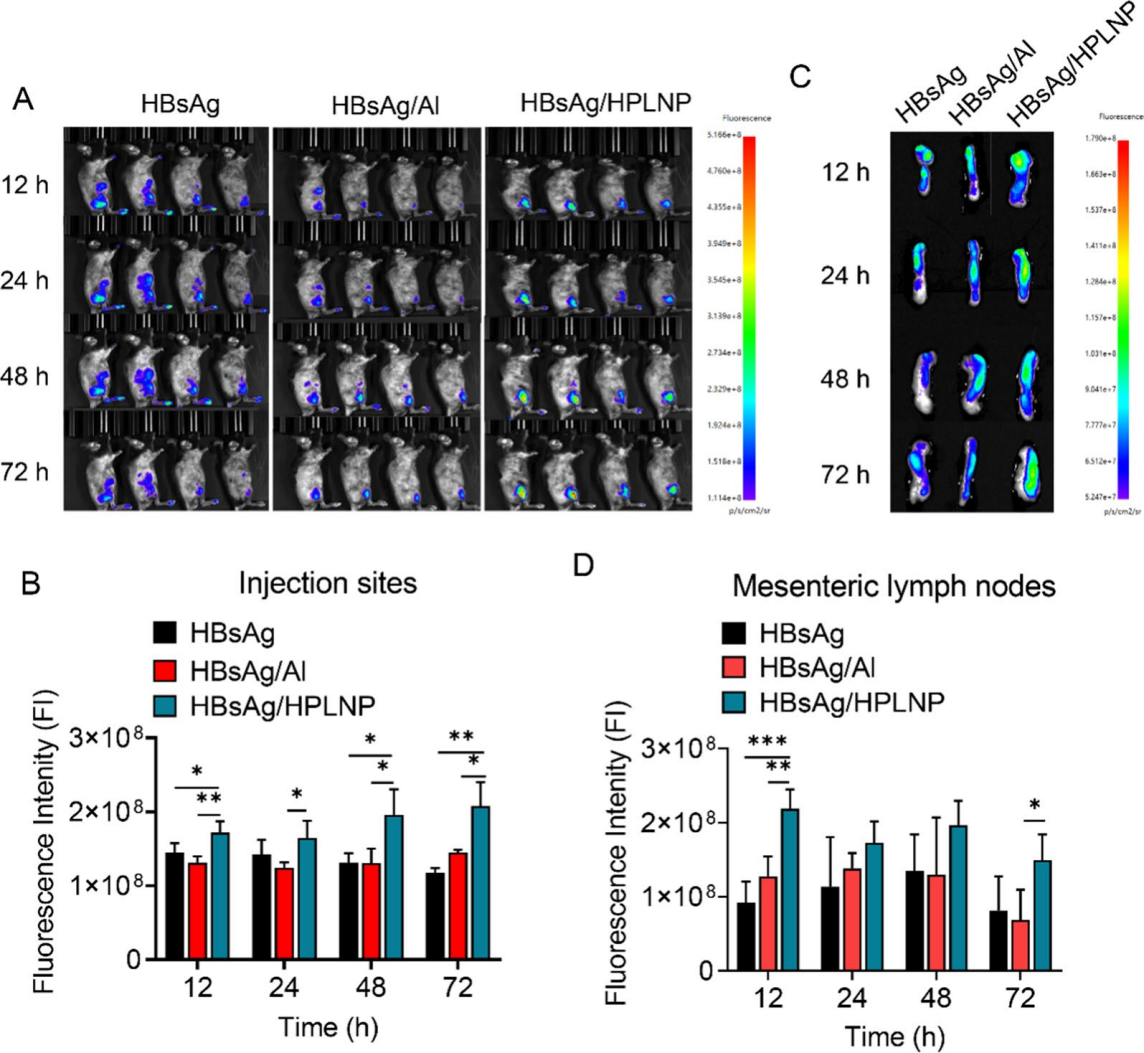
**A** The fluorescence images and **B** the relative fluorescence intensity (FI) of the injection site in mice after injecting with HBsAg, HBsAg/Al and HBsAg/HPLNP formulations at a concentration of 1 µg HBsAg per mouse, for varying time periods. **C** The fluorescence images and **D** the FI of mesenteric lymph nodes after injecting with HBsAg, HBsAg/Al and HBsAg/HPLNP formulations at a concentration of 1 µg HBsAg per mouse, for varying time periods. Tukey's multiple comparisons test was used for statistical analyse

One possible mechanism by which adjuvants improve the immune response is by prolonging the retention of antigen at the injection site and enhancing lymph node drainage. Images obtained showed varying rates of antigen diffusion at the injection site among the HBsAg, HBsAg/Al, and HBsAg/HPLNP groups (Fig. 4A). The HBsAg group exhibited faster antigen diffusion compared to the HBsAg/Al and HBsAg/HPLNP groups, as evidenced by a larger fluorescence area after injection for 12 h. The HBsAg/HPLNP group retained the antigen at the injection site for up to 72 h with a much lower diffusion speed than that in HBsAg group, suggesting that HPLNPs effectively prolonged the antigen depot and reduced antigen diffusion. While fluorescence signals were still detectable at the injection site of HBsAg and HBsAg/Al groups 72 h post-injection, and their mean fluorescence intensities were significantly lower compared to the HBsAg/HPLNP group (as shown in Fig. 4B). Within 12 to 72 h after the injection, the MFI of the HBsAg group decreased slowly (from 1.45 × 10^8^ to 1.17 × 10^8^) and the HBsAg/Al group showed little change (around 1.31–1.45 × 10^8^). While the MFI of the HBsAg/HPLNP group at 72 h (2.08 × 10^8^) was even higher than that at 12 h (1.72 × 10^8^). This finding may be attributed to the strong antigen depot effect of HPLNPs, which efficiently condensed the antigen at the injection site and released it slowly over time. The observed increase in fluorescence was likely due to the gradual release of the antigen from the injected muscle site, further indicating that HPLNPs enhance and prolong the antigen depot effect, which is beneficial for enhancing the antigen uptake by APCs and the following immune response.

Meanwhile, the fluorescence of the lymph node in HBsAg/HPLNP group at 12 h was obviously higher than that of the HBsAg (**p < 0.01) or HBsAg/Al (***p < 0.001) group, indicating a faster and stronger antigen drainage to the mesenteric lymph node (Fig. 4C). On the other hand, there was a reduction in the signal observed at the lymph nodes after 48 h, which was most likely due to the absorption of the vaccine nanoparticles into the lymphatic capillaries in the subcutaneous tissue. These nanoparticles were then transported into the lymph nodes, where the HBsAg could have been taken up by lymphocytes and broken down by the APCs. Over time, the antigen would be distributed throughout the circulatory and lymphatic system, resulting in a decrease in the signal observed at the lymph nodes (Fig. 4D). Despite a decrease in fluorescence intensity over time, the HBsAg/HPLNP group exhibited much higher fluorescence intensity in the lymph nodes compared to the HBsAg/Al group after 72 h (*p < 0.05). These results provide evidence for the advantages of HPLNP as an adjuvant, which include prolonging the antigen depot at the injection site, enhancing antigen lymph node drainage, and decreasing antigen diffusion. Moreover, the degradation of active vaccine constituents can result in functional loss, primarily attributed to enzymatic degradation causing a decline in antigen effectiveness. Al adjuvants aid in diminishing antigen degradation via the "antigen depot effect", curtailing antigen release kinetics and minimizing interaction with endogenous enzymes [57]. Similarly, electrostatic interactions facilitated the successful absorption of HBsAg-VLP antigen onto HPLNP, yielding a stable nanoformulation supported by DLS data in Fig. 2A, B. Hence, the HPLNP-induced "antigen depot effect" might also attenuate HBsAg degradation. Furthermore, PEGylation, whether via chemical conjugation or nanoparticle encapsulation, has been demonstrated to heighten protein colloidal stability in numerous investigations [58, 59]. Drawing from our prior findings, nanoparticle PEGylation concurrently reduces serum protein adsorption [52, 53], thereby mitigating enzyme-HBsAg interactions. Consequently, the PEGylated HBsAg/HPLNP formulation could mitigate HBsAg enzymatic degradation.

### Activation of lymphocytes in lymph node

**Fig. 5:**
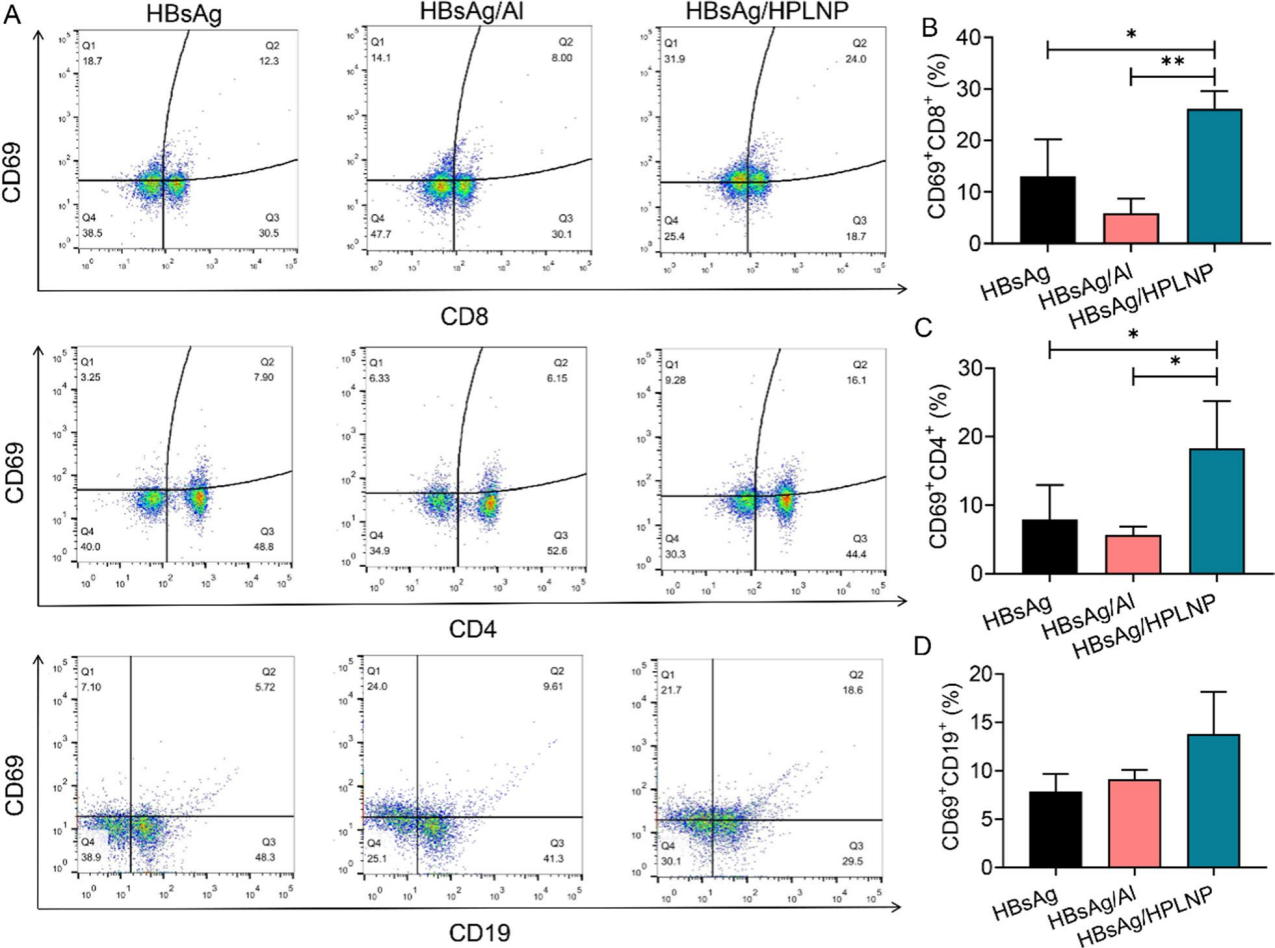
**A** Flow cytometry graphs and **B** quantitative analysis of CD69 + CD8 + T cells **C** CD69 + CD4 + T cells and (**D**) CD69 + CD19 + B cells in mesenteric lymph nodes after the first immunisation with different formulations for 12 h. One-way ANOVA was used for statistical analyse

Encouraged by the antigen depot and lymph node drainage outcome, we further explored the adjuvant effects of HPLNPs vaccines on aspects of in vivo lymphocytes activation. After the first immunisation with different formulations for 18 h, the lymphocytes in mesenteric lymph nodes were analysed by flow cytometry (Fig. 5A). It revealed that the HBsAg/HPLNP group induced 26.1% of CD69 + CD8 + T cells, which was significantly higher than the HBsAg (~ 13.02%, *p < 0.05) or HBsAg/Al (~ 5.87%, **p < 0.01) groups (Fig. 5B). Meanwhile, similar trend can be found in CD69 + CD4 + T cells among these groups (Fig. 5C). There was no significant difference in activation of B cells among these groups (Fig. 5D). It’s encouraging that the HPLNPs showed superiority in activating T cells in lymph nodes compared with commercial Al adjuvant.

### In vivo immunisation

**Fig. 6:**
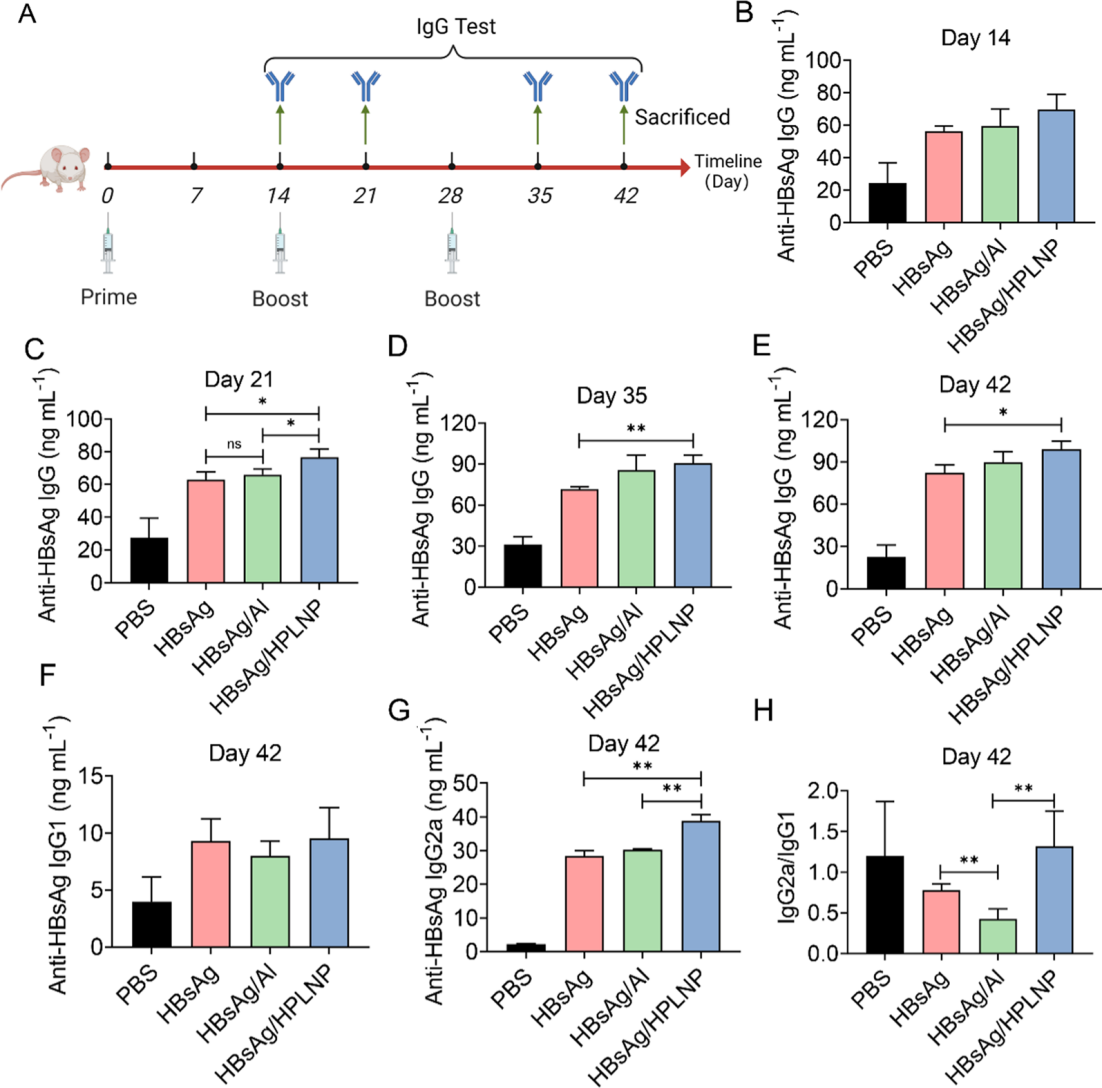
**A** Schematic timeline illustration of the in vivo immunisation and the serum anti-HBsAg IgG test. **B** Serum anti-HBsAg IgG concentrations after the prime immunisation with different formulations at the HBsAg concentration of 1 µg/leg (at the equivalent dose of 2 µg/mouse) on Day 14. **C** Serum anti-HBsAg IgG concentrations after the boost with different formulations at the HBsAg concentration of 1 µg/leg on Day 21. **D** Serum anti-HBsAg IgG concentrations after the second boost with different formulations at the HBsAg concentration of 1 µg/leg on Day 35 or **E** on Day 42, respectively. **F** The serum anti-HBsAg IgG1 concentrations, **G** anti-HBsAg IgG2a concentrations and (**H**) the anti-HBsAg IgG2a/IgG1 concentration ratios after the second boost with different formulations at the HBsAg concentration of 1 µg/leg on Day 42, respectively. One-way ANOVA was used for statistical analyse

Based on the promising results of cellular uptake and lymph node drainage, an in vivo immunization study was conducted using the schematic procedure shown in Fig. 6A. The production of anti-HBsAg IgG tended to increase over time after the three intramuscular injections. The HBsAg/HPLNP group had considerably higher anti-HBsAg IgG concentration than the HBsAg and HBsAg/Al groups after the prime immunisation on Day 14 (Fig. 6B). After the first boost, the HBsAg/HPLNP group induced significantly higher anti-HBsAg IgG level compared to the HBsAg alone (*p < 0.05) or HBsAg/Al group (*p < 0.05) on Day 21 (Fig. 6C). After the second boost, the anti-HBsAg IgG concentration of the HBsAg/HPLNP group was still significantly higher than that of the HBsAg group on Day 35 (**p < 0.01, Fig. 6D) and Day 42 (*p < 0.05, Fig. 6E). These results indicated that the HPLNP adjuvant can induce a quicker and stronger humoral immune response (Th2 type) than the pure HBsAg group and Al adjuvant.

Furthermore, a preferred adjuvant should promote both Th1 and Th2 responses. The Th1-type immune response is particularly important especially in chronic virus-related diseases like Hepatitis B virus infection. However, the frequently used Al adjuvants are biased towards a Th2-driven response. Then we analysed the anti-HBsAg IgG2a/IgG1 ratio to evaluate the ability of HPLNPs adjuvant for inducing a Th1-driven response. In Fig. 6F, the concentrations of anti-HBsAg IgG1 isotype related to the humoral response (Th2) were similar in all groups after three injections on Day 42. However, the anti-HBsAg IgG2a reflecting the T cell activation (Th1) in HBsAg/HPLNPs group was significantly higher than that in HBsAg (** *p* < 0.01) or HBsAg/Al group (**p < 0.01) (Fig. 6G). The anti-HBsAg IgG2a/IgG1 ratio of HBsAg/HPLNP group was much higher than HBsAg group or HBsAg/Al group (**p < 0.01), while the HBsAg/Al group even reduced the IgG2a/IgG1 ratio compared to the pure HBsAg in Fig. 6H (**p < 0.01). These results prove that the HPLNP is effective than Al adjuvant in stimulating the Th1-driven immune response, which correlates to the cytotoxic T lymphocyte (CTL) reactions.

**Fig. 7:**
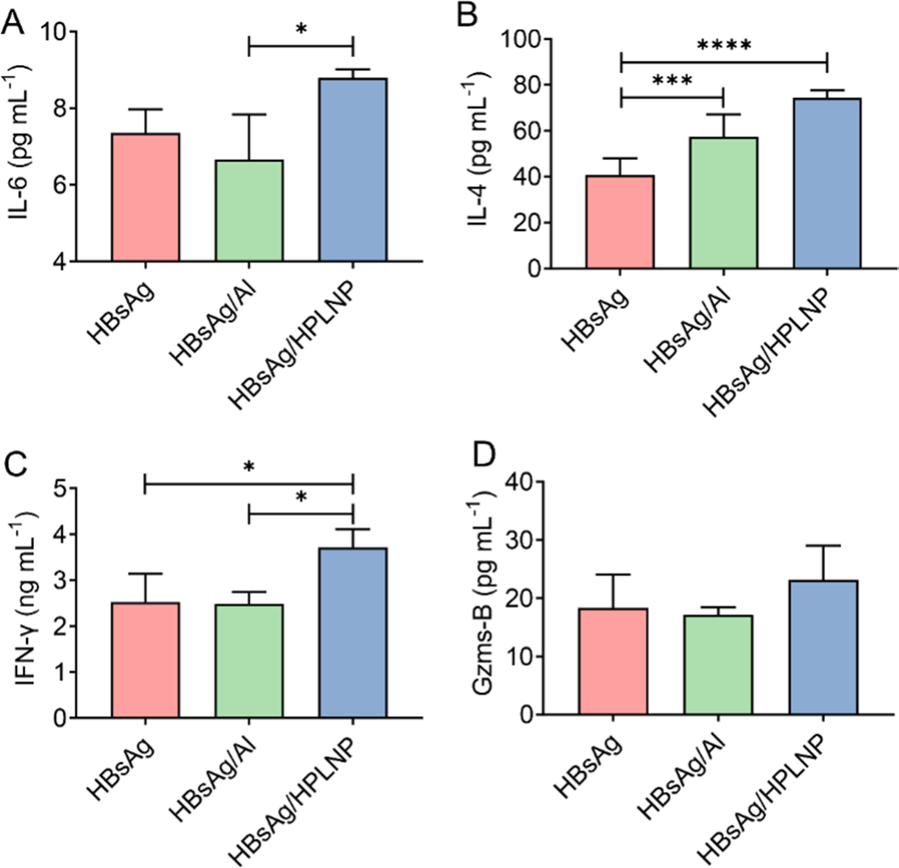
**A** The serum IL-6, **B** IL-4, **C** IFN-γ and **D** Gzms-B concentration after the second boost of different vaccine formulations on Day 42 in mice. One-way ANOVA was used for statistical analyse

Except for the antibody analysis, we also tested the serum cytokine secretion. In Fig. 7A, the secretion of IL-6 in HBsAg/HPLNP group was obviously higher than that in HBsAg/Al group (*p < 0.05), which confirmed the promotion of humoral immunity by HPLNPs, as IL-6 plays an important role in B cell proliferation, differentiation and antibody production. Also, both the HBsAg/HPLNP and HBsAg/Al groups had a much higher IL-4 production than the HBsAg group, which further confirmed the HPLNPs can induce a Th2-driven response as effectively as Al adjuvant (Fig. 7B). Meanwhile, the HPLNPs significantly enhanced the serum concentration of IFN-γ than the HBsAg or HBsAg/Al groups, emphasizing the strong Th1 driving effect (Fig. 7C). It was because IFN-γ was the typical cytokine secreted by Th1 or CD8 + T cells. This result made a good correlation with the IgG2a/IgG1 results in Fig. 6H. Besides, the cytokine of Gzms-B was also analysed (Fig. 7D). Taken together, the HPLNP adjuvant can effectively improve the Th2 immune responses but also has a strong propensity to induce the Th1 immune response, which was more advantageous than the aluminium adjuvant. Antigens typically degrade within the acidic and enzymatic environment of endosomes, generating short peptide fragments. These peptides subsequently interact with MHC-II molecules, forming MHC-II-antigen complexes transported to the cell surface, engaging CD4 + T cells for humoral immune responses. Exogenous antigens can access the MHC-I pathway through cytoplasmic processing and cross-presentation, wherein APCs process antigens into short peptides presented to CD8 + T cells, activating cellular immunity [60]. This cross-presentation is pivotal for robust immune responses against intracellular pathogens. Positively charged HPLNP induces a "proton sponge effect", aiding endosomal escape and antigen cytoplasmic delivery. Thus, this potentially leads to MHC-I-antigen complex formation, fostering cellular immunity activation.

### Intracellular cytokines secretion, activation and proliferation of splenocytes

**Fig. 8:**
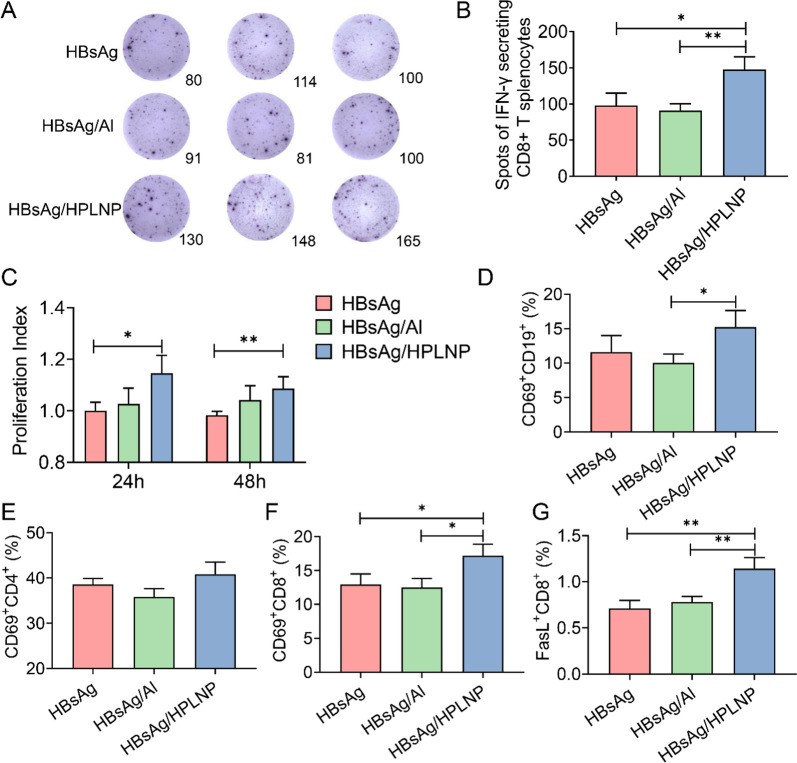
**A** Representative spots and **B** quantitative analysis of IFN-γ secreting CD8 + T splenocytes. **C** In vitro splenocytes proliferation measured by CCK-8 after restimulation by 5 µg mL^−1^ of HBsAg for 24 or 48 h. **D** CD69 positive cells among CD19 + B cells, (**E**) CD4 + T cells, (**F**) CD8 + T cells, for characterisation of activation measured by flow cytometry. **G** FasL + cells in CD8 + T cells for characterisation of cytotoxic activity detected by flow cytometry. Data are all expressed as Means ± SD. One-way ANOVA was used for statistical analyse

As the spleen is the most significant immune organ, we evaluated the activation of splenocytes and the intracellular cytokine secretion. After restimulated with HBsAg for 48 h, the number of IFN-γ secreting CD8 + T splenocytes in HBsAg/HPLNP group was significantly higher than that of the HBsAg or HBsAg/Al groups, indicating a preference for cellular immune response (Fig. 8A). For instance, a maximum of 1.5-fold or 1.67-fold of spots number could be observed for the HBsAg/HPLNP group, in comparison to the HBsAg group (*p < 0.05) or HBsAg/Al group (**p < 0.01), respectively (Fig. 8B). Furthermore, we observed a significantly higher antigen-specific proliferation of splenocytes in the HBsAg/HPLNP group than that in the HBsAg group at both 24 h (*p < 0.05) and 48 h (**p < 0.01) post-re-stimulation with HBsAg antigen (Fig. 8C). Meanwhile, the activation of splenocytes including B cell and T cell were also analysed. The percentage of CD69 + CD19 + B cells in HBsAg/HPLNP group was obviously higher than that of Al adjuvant in Fig. 8D (*p < 0.05). No significant difference was observed in the CD69 + /CD4 + T cells among these groups (Fig. 8E). However, both the CD69 + /CD8 + T and FasL + /CD8 + T cells in the HBsAg/HPLNP group were significantly increased compared to the HBsAg or the HBsAg/Al group, respectively (Fig. 8F, G). It confirmed that the HBsAg/HPLNP group had great advantages in activating the cytotoxic T cells. Taken together, these results demonstrated the superior ability of HPLNP in potentiating both humoral immunity and cellular immunity.

### Safety assessment

**Fig. 9:**
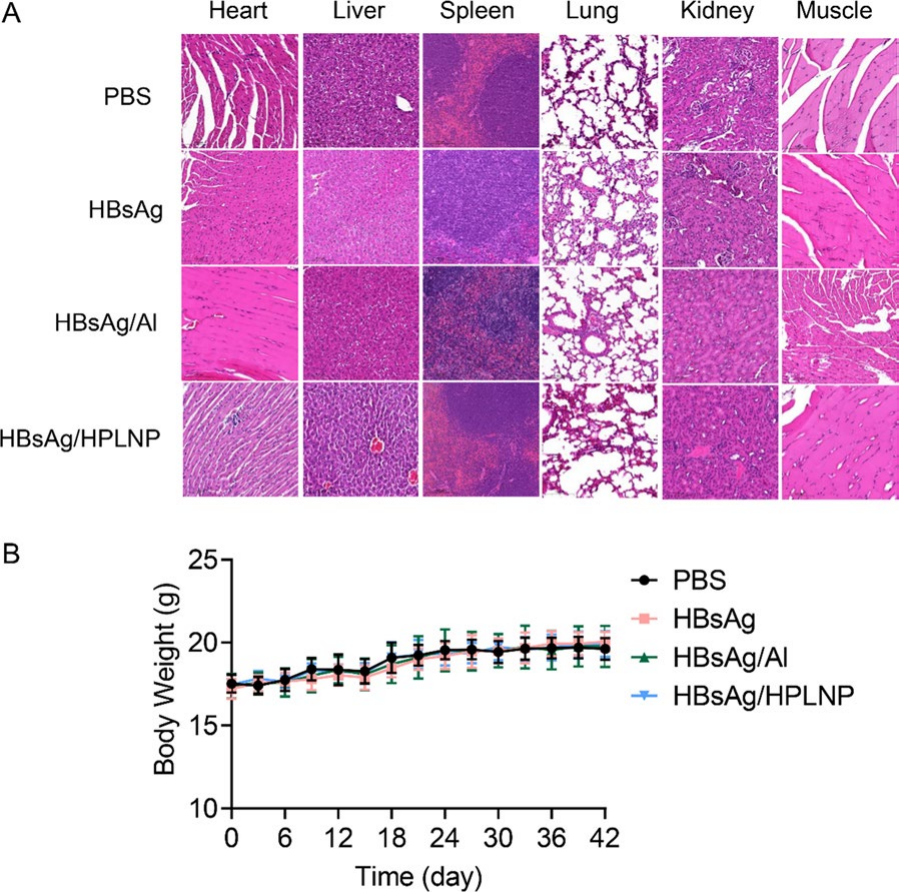
**A** Immunohistochemical staining of different organs and intramuscular injection site after prime immunisation with different vaccines for 24 h. **B** Mice body weights during the immunisation with different vaccine formulations

To evaluate the biocompatibility of the HBsAg/HPLNP formulation, the immunohistochemistry of important organs and the intramuscular injection site were evaluated after the first immunisation for 24 h (Fig. 9A). Compared with the PBS group, there was no significant tissue damage on the main organs and the injected muscle site among the HBsAg, HBsAg/Al and HBsAg/HPLNP groups. It revealed that HPLNPs have good biocompatibility and limited side effects as a novel vaccine adjuvant. During the immunisation, the mice body weights of all these groups were also analysed to evaluate the safety of vaccine formulations. The negligible body weight change in HBsAg/HPLNP group compared to the PBS group further confirmed the satisfying biosafety of HPLNPs (Fig. 9B).

## Conclusion

Here we developed a novel hybrid polymer lipid nanoparticle (HPLNP) as the HBsAg-VLP vaccine adjuvant. The HPLNP was composed of two FDA-approved materials, and can be prepared by a simple one-pot method based on several minutes of mixing, stirring and organic solvent evaporation. The optimised formulation with the mass ratio of HBsAg/HPLNP = 1/600 significantly improved the antigen cellular uptake by APCs. The optimised HBsAg/HPLNP formulation largely enhanced the antigen depot effect and prolonged the duration of antigen at the injection site. The lymph node drainage of antigen assisted by HPLNPs was obviously faster and higher compared to the use of Al adjuvant. Therefore, the serum anti-HBsAg IgG concentration of HBsAg/HPLNP group was much higher than the pure HBsAg group or HBsAg/Al group after the boost immunisation in mice. Even after the second boost, the final anti-HBsAg IgG production of the HBsAg/HPLNP group was obviously higher than the HBsAg group. Except for the effective humoral immunity, the markedly improved IgG2a/IgG1 of HPLNP group indicated a strong propensity for HPLNP to induce the Th1-driven immune response. Meanwhile, the elevated IL-4 and IL-6 secretion of HBsAg/HPLNP group further confirmed the improved humoral immunity as they were important in the B cell proliferation, differentiation and antibody production. Furthermore, the HPLNP enhanced the production of IFN-γ and FasL + /CD8^+^ T cells, which was obviously higher than the commercial Al adjuvant, emphasising the strong cellular immune stimulating effect. Last but not the least, there was limited systematic toxicity during the whole immunization in the HPLNPs group, demonstrated by the immunohistochemistry and mice body weights evaluation. Therefore, our newly developed HPLNP adjuvant, which offers advantages including good biocompatibility, easy preparation, low cost and the ability to improve both humoral and cellular immunity, would be a potential alternative to the aluminium adjuvant.

### Supplementary Information

**Additional file 1: Table S1.** The size distribution, PDI and zeta potentials of HBsAg, HPLNP and HBsAg/HPLNP (w/w=1/600) formulation. **Table S2.** The size distribution, PDI and zeta potentials of HBsAg/HPLNP formulations with different HBsAg to HPLNP mass ratios. **Figure S1.** The UV absorption curve of HBsAg, HPLNP, HBsAg/HPLNP and the HBsAg/HPLNP after storage at 4 ^o^C for 7 days. The dashed line represents the curve obtained by summing the absolute values of HBsAg and HPLNP sample**. Figure S2.** (A) The size distribution and (B) zeta potential of the freshly prepared HBsAg/HPLNP (w/w=1/600) formulation and the HBsAg/HPLNP (w/w=1/600) formulation after storage at 4 ^o^C for 7 days. **Figure S3.** TEM of the HBsAg/HPLNP (w/w=1/600) formulation (Bar=100 nm). **Figure S4.** (A) Histogram and (B) quantitative assessment of MHC-II positive cell percentage after 24-hour incubation with HBsAg, HBsAg/Al or HBsAg/HPLNP formulation at a concentration of 0.5 μg mL^-1^ of HBsAg on RAW264.7 cells by flow cytometry (Sparrow, China). **Figure S5.** Cell viability of mouse spleen lymphocyte following a 24-hour incubation with HBsAg, HBsAg/Al or HBsAg/HPLNP formulations at various HBsAg concentrations.

## Data Availability

All data generated or analysed during this study are included in this published article and its supplementary information files, while the source data can be obtained from the corresponding author upon a reasonable request.
