# Supplementary material for: Potentiating humoral and cellular immunity using a novel hybrid polymer-lipid nanoparticle adjuvant for HBsAg-VLP vaccine
Source: J Nanobiotechnology. 2023 Nov 22;21:441. doi: 10.1186/s12951-023-02116-6 (PMC10666313; doi:10.1186/s12951-023-02116-6)
Supplement: Supplementary file 1 — Additional file 1: Table S1. The size distribution, PDI and zeta potentials of HBsAg, HPLNP and HBsAg/HPLNP (w/w=1/600) formulation. Table S2. The size distribution, PDI and zeta potentials of HBsAg/HPLNP formulations with different HBsAg to HPLNP mass ratios. Figure S1. The UV absorption curve of HBsAg, HPLNP, HBsAg/HPLNP and the HBsAg/HPLNP after storage at 4 oC for 7 days. The dashed line represents the curve obtained by summing the absolute values of HBsAg and HPLNP sample. Figure S2. (A) The size distribution and (B) zeta potential of the freshly prepared HBsAg/HPLNP (w/w=1/600) formulation and the HBsAg/HPLNP (w/w=1/600) formulation after storage at 4 oC for 7 days. Figure S3. TEM of the HBsAg/HPLNP (w/w=1/600) formulation (Bar=100 nm). Figure S4. (A) Histogram and (B) quantitative assessment of MHC-II positive cell percentage after 24-hour incubation with HBsAg, HBsAg/Al or HBsAg/HPLNP formulation at a concentration of 0.5 μg mL-1 of HBsAg on RAW264.7 cells by flow cytometry (Sparrow, China). Figure S5. Cell viability of mouse spleen lymphocyte following a 24-hour incubation with HBsAg, HBsAg/Al or HBsAg/HPLNP formulations at various HBsAg concentrations. [file 12951_2023_2116_MOESM1_ESM.docx]

**Additional file 1**

Potentiating humoral and cellular immunity using a novel hybrid polymer-lipid nanoparticle adjuvant for HBsAg-VLP vaccine

Xuhan Liu^1^, Qiuxia Min^2^, Huiping Song^1,3^, Aochun Yue^1,4^, Qin Li^1^, Qing Zhou^5*^, Wei Han^1*^

^1^Department of Emergency Medicine, Shenzhen University General Hospital, Shenzhen University Clinical Medical Academy, Shenzhen University, No. 1098 Xueyuan Avenue, Shenzhen 518000, PR China.

^2^Department of Pharmacy, First People’s Hospital of Yunnan Province, Kunming University of Science and Technology, No. 157 Jinbi Road, Kunming, 650034, Yunnan, China

^3^First School of Clinical Medicine, Shandong University of Traditional Chinese Medicine, Jinan, China

^4^Centre of Integrated Chinese and Western Medicine, School of Clinical Medicine, Qingdao University, Qingdao, China

^5^The Center for Biomedical Research, Tongji Hospital, Tongji Medical College, Huazhong University of Science and Technology, Wuhan, China

**** To whom correspondence should be addressed.***

*Qing Zhou: The Center for Biomedical Research, Tongji Hospital, Tongji Medical College, Huazhong University of Science and Technology, Wuhan, China. Email:zhouqing@tjh.tjmu.edu.cn*

*Wei Han: Department of Emergency Medicine, Shenzhen University General Hospital, Shenzhen University Clinical Medical Academy, No. 1098 Xueyuan Avenue, Shenzhen 518000, Guangdong Province, China. Email: sugh_hanwei@szu.edu.cn*

| **Sample** | **Size (d.nm)** | **PDI** | **Zeta potential (mV)** |
| --- | --- | --- | --- |
| HBsAg | 35.9±0.8 | 0.34±0.02 | -17.7±1.6 |
| HPLNP | 53.3±0.2 | 0.32±0.01 | 27.9±1.7 |
| HBsAg/HPLNP (w/w=1/600) | 56.7±0.3 | 0.21±0.03 | 33.6±0.7 |

Table S1. The size distribution, PDI and zeta potentials of HBsAg, HPLNP and HBsAg/HPLNP (w/w=1/600) formulation.

| **HBsAg/HPLNPs (w/w)** | **1/20** | **1/50** | **1/100** | **1/200** | **1/300** | **1/400** | **1/600** | **1/800** | **1/1600** |
| --- | --- | --- | --- | --- | --- | --- | --- | --- | --- |
| **Size (nm)** | 75.3±10.2 | 69.9±9.8 | 74.3±12.3 | 60.8±0.1 | 60.3±0.2 | 59.4±0.3 | 56.7±0.3 | 57.1±0.4 | 57.6±1.0 |
| **PDI** | 0.33±0.02 | 0.32±0.03 | 0.31±0.05 | 0.28±0.03 | 0.24±0.03 | 0.28±0.02 | 0.21±0.03 | 0.32±0.02 | 0.34±0.02 |
| **Zeta potential (mV)** | 27.4±4.9 | 36.6±3.5 | 17.1±2.0 | 24.1±1.7 | 26.5±2.6 | 29.6±1.5 | 33.6±0.7 | 37.2±5.7 | 31.7±0.7 |

Table S2. The size distribution, PDI and zeta potentials of HBsAg/HPLNP formulations with different HBsAg to HPLNP mass ratios.

Figure S1. The UV absorption curve of HBsAg, HPLNP, HBsAg/HPLNP and the HBsAg/HPLNP after storage at 4^o^C for 7 days. The dashed line represents the curve obtained by summing the absolute values of HBsAg and HPLNP sample.


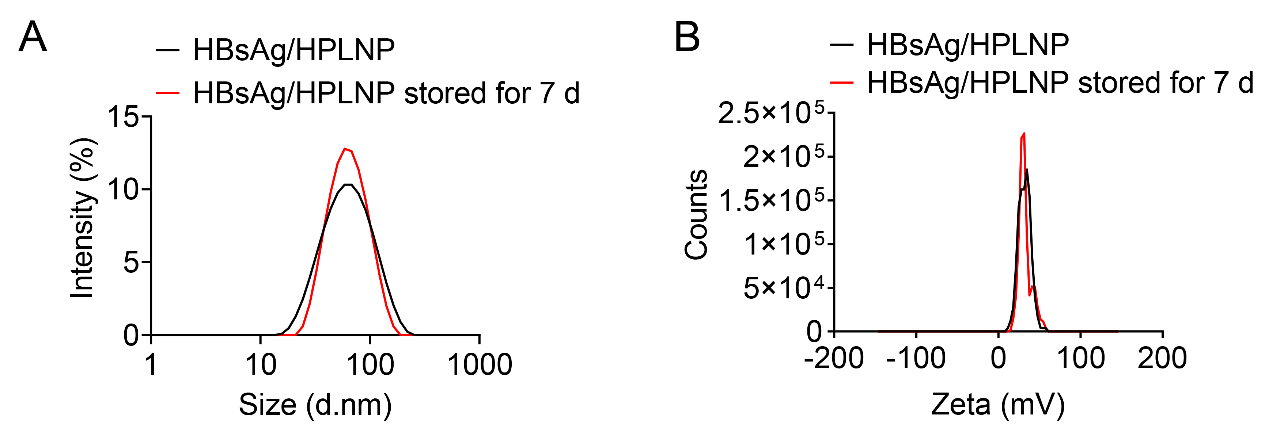


Figure S2. (A) The size distribution and (B) zeta potential of the freshly prepared HBsAg/HPLNP (w/w=1/600) formulation and the HBsAg/HPLNP (w/w=1/600) formulation after storage at 4^o^C for 7 days.


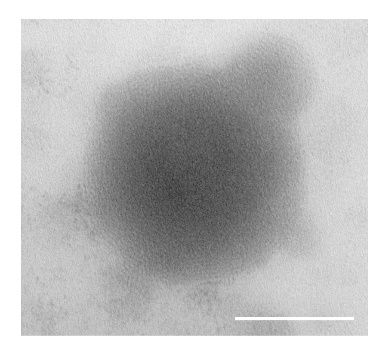


Figure S3. TEM of the HBsAg/HPLNP (w/w=1/600) formulation (Bar=100 nm).


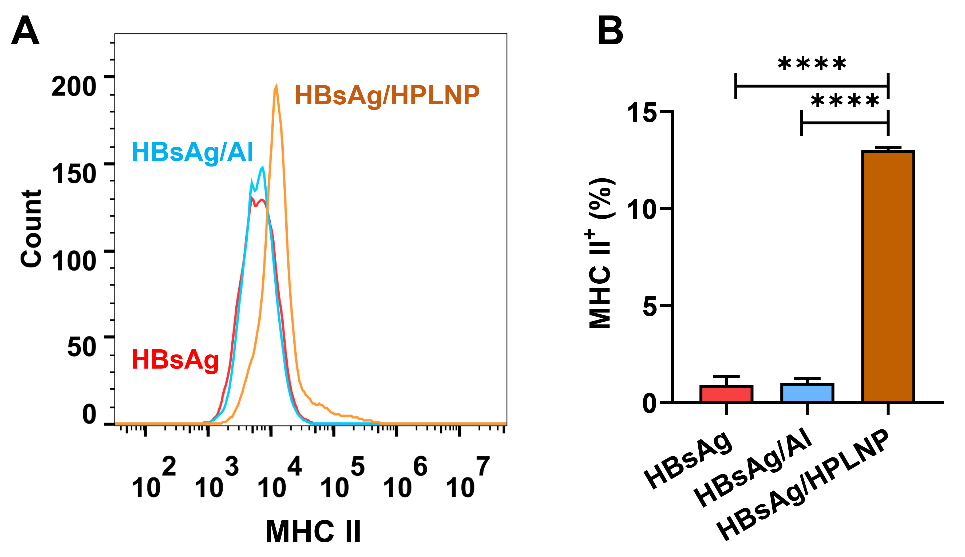


Figure S4. (A) Histogram and (B) quantitative assessment of MHC-II positive cell percentage after 24-hour incubation with HBsAg, HBsAg/Al or HBsAg/HPLNP formulation at a concentration of 0.5 μg mL^-1^ of HBsAg on RAW264.7 cells by flow cytometry (Sparrow, China).


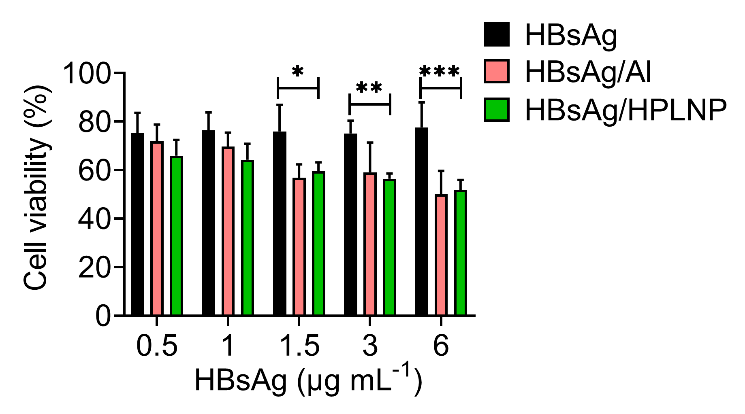


Figure S5. Cell viability of mouse spleen lymphocyte following a 24-hour incubation with HBsAg, HBsAg/Al or HBsAg/HPLNP formulations at various HBsAg concentrations.
